# Assessing the JEOL CRYO ARM 300 for high-throughput automated single-particle cryo-EM in a multiuser environment

**DOI:** 10.1107/S2052252520006065

**Published:** 2020-06-11

**Authors:** Marcus Fislage, Alexander V. Shkumatov, Annelore Stroobants, Rouslan G. Efremov

**Affiliations:** a Center for Structural Biology, Vlaams Instituut voor Biotechnologie, Pleinlaan 2, Brussels 1050, Belgium; b Structural Biology Brussels, Department of Bioengineering Sciences, Vrije Universiteit Brussel, Pleinlaan 2, Brussels 1050, Belgium

**Keywords:** automated single-particle data collection, high-resolution 3D reconstruction, cold FEG

## Abstract

Single-particle cryo-EM has become an indispensable structural biology method. It requires regular access to high-resolution electron cryogenic microscopes. To fully utilize the capacity of the expensive high-resolution instruments, the time used for data acquisition and the rate of data collection have to be maximized. This in turn requires high stability and high uptime of the instrument. One of the first 300 kV JEOL CRYO ARM 300 microscopes has been installed at the cryo-EM facility BECM at VIB-VUB, Brussels, where the microscope is used for continuous data collection on multiple projects. Here, the suitability and performance of the microscope is assessed for high-throughput single-particle data collection. In particular, the properties of the illumination system, the stage stability and ice contamination rates are reported. The microscope was benchmarked using mouse heavy-chain apoferritin which was reconstructed to a resolution of 1.9 Å. Finally, uptime and throughput statistics of the instrument accumulated during the first six months of the facility operation in user access mode are reported.

## Abstract 2

This paper presents a detailed assessment of the performance of the CRYO ARM 300 electron microscope in a multiuser cryo-EM facility setting. It is shown that the microscope can be used for reliable automated data collection. The strengths and weaknesses of the hardware are discussed.

## 1. Introduction   

As cryo-EM is becoming the major tool for structural biology (Kühlbrandt, 2014 ▸), the demand for microscope time has increased significantly in the last few years. Access to high-resolution microscopes often presents a bottleneck in structural biology projects. This is particularly true for difficult projects, where regular high-resolution data collection is required. The efforts to accelerate and facilitate structural projects include establishing a constantly growing number of cryo-EM facilities with supervised access to automated data collection, increasing throughput of data collection (Cheng *et al.*, 2018 ▸; Wu *et al.*, 2019 ▸), designing cheaper and better screening microscopes (Naydenova *et al.*, 2019 ▸), and improving sample preparation methodologies (Ravelli *et al.*, 2019 ▸; Razinkov *et al.*, 2016 ▸).

The increase in data collection throughput on high-resolution 300 kV microscopes has been achieved by enabling fast sample pre-screening using automated grid loading systems and development of automated data collection software (Mastronarde, 2005 ▸; Suloway *et al.*, 2005 ▸) which permits continuous data collection over periods of days with minor operator supervision.

The Titan Krios electron microscope (Thermo Fisher Scientific) became the reference microscope for high-resolution single-particle cryo-EM. At the Biological Electron Cryogenic Microscopy (BECM) facility at VUB/VIB, Brussels, one of the first new-generation cryogenic 300 kV microscopes from JEOL, the CRYO ARM 300, has been installed.

The microscope is equipped with a 300 kV cold FEG (cFEG), four-lens condenser illumination system, high-contrast objective lens, autoloader for 12 cryo-grids, phase plate, in-column Ω energy filter and K2 direct electron detector (Gatan). In this configuration, the microscope has been used for a period of six months of continuous user access and high-throughput data collection. Although the suitability of the CRYO ARM 300 has been reported for collection of high-resolution data (Hamaguchi *et al.*, 2019 ▸), the stability and performance of the microscope in the setting of a multiuser facility operating in a mode of continuous single-particle data collection is not known.

Here, we assess the performance of the CRYO ARM 300 during single-particle cryo-EM data collection in the setting of a multiuser facility. We evaluate stability of the cFEG, properties of the cryo-stage, ice contamination rates and the performance of the microscope during extended automated single-particle data collection. We also provide statistics on the uptime of the microscope and data collection throughput obtained during a period of six months of continuous microscope operation.

## 2. Materials and methods   

### 2.1. Measurements of cFEG flux and beam tilt   

The intensity of the electron beam was probed at regular intervals by measuring average counts of the images recorded on a K2 camera without sample. The measurements were performed automatically using *SerialEM* (v. 3.7.6; Mastronarde, 2005 ▸). Gun flashing with the LOW setting was performed every 4.5 h, except for one point at which the HIGH flash setting was used. The beam tilt was estimated using coma-free alignment by the CTF procedure of *SerialEM*. Before the measurement, the stage was moved over a continuous carbon region on the grid and stabilized for 1 min.

### 2.2. Stage stability measurements   

To estimate the drift after liquid nitro­gen refilling, both the cryo-stage and the autoloader dewars were refilled simultaneously. Upon termination of the refill, movies composed of 10 frames with 0.2 s exposure per frame were recorded at a nominal magnification of 60 000 on a K2 detector. The images were recorded every 30 s for 30 min.

To evaluate the drift rate after grid exchange, a grating replica grid (EMS Diasum) was loaded onto the stage from the autoloader storage. Images were recorded at a magnification of 60 000 on a K3 detector at intervals of 30 s for a period of 30 min. A total of 100 movie frames with per frame exposure of 0.038 s were acquired for each time point. In both cases the trajectory of the sample movement was determined by movie frame alignment in *MotionCor2* (Zheng *et al.*, 2017 ▸).

To calculate the drift, movie frames corresponding to late exposure (starting from a dose of 4 e Å^−2^, as defined in *RELION2.0* and later) until the end of exposure were used. The total drift was estimated either by measuring the distance between the first late exposure frame and the last frame (referred to as the end-to-end method) or by summing up the absolute shifts between consecutive frames (referred to as the integral path method). The specific method used is indicated in the figure legends and the choice of method is explained in Section 3.1.2.

For the single-particle dataset of apoferritin, 80 movie frames with an exposure time of 0.1 s per frame were collected per micrograph on a K2 camera.

### 2.3. Ice thickness measurements   

Ice thickness was measured using the energy filter method (Rice *et al.*, 2018 ▸). The EM images were recorded with the inserted slit set to a width of 20 eV, immediately followed by an image with a retracted slit. The thickness was calculated as
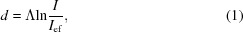
where *I* and *I*_ef_ are beam intensities without and with the energy filter slit inserted, respectively, and Λ is the apparent mean-free path of electrons in ice. We used the value of 400 nm for Λ estimated by Rice *et al.* (2018 ▸) for the Titan Krios microscope and by theoretical calculations (Vulović *et al.*, 2013 ▸) as a rough estimate for the CRYO ARM 300. The ratio of intensities without and with an energy filter, *I* and *I*_ef_, were measured as ratios of averaged intensities of images recorded on a K2 detector for in-column contamination rate measurements and on a K3 detector for contamination rate measurements in the autoloader. In both cases images were acquired in electron counting mode.

The ice contamination in the column was measured during data collection of apoferritin using the energy filter method for every 20th image. To assess the ice contamination in the autoloader, the ice thickness in selected areas was measured after grid insertion in the microscope and on days 7, 12 and 22 thereafter. In between measurements, the grid was stored in the autoloader. The grid was aligned to the same area using the *SerialEM* function *AlignToItem*. The ice thickness was measured by the energy filter method at a magnification of 5000. The electron dose per image was 0.06 e^−^ Å^−2^. The average ice thickness is reported from the area within each hole. The measurements were performed for 15 holes at each time point.

### 2.4. Automated data collection   

Automated data collection was performed using *SerialEM* (v. 3.7.6). The *SerialEM* scripts initially provided by JEOL application scientists were significantly modified and extended to allow for versatile scenarios of data collection including sparse focusing and ice thickness measurements (see the supporting information).

### 2.5. Purification and preparation of cryo-EM samples of apoferritin   

The plasmid with mouse heavy-chain apoferritin was supplied by Dr H. Yanagisawa at the University of Tokyo. After transformation of the plasmid in BL21 *E. coli*, cells were grown in Luria–Bertani medium at 37°C, with 0.5% glucose and 50 µg ml^−1^ kanamycin, to an OD at 600 nm of 0.5. The protein expression was induced with 1 m*M* IPTG (Inalco) for 4 h. Subsequent steps were all performed at 4°C. The cells from a 1 l culture were harvested by centrifugation at 6500*g* for 15 min and resuspended in 20 ml lysis buffer (30 m*M* HEPES–NaOH pH 7.5, 300 m*M* NaCl, 1 m*M* MgSO_4_), supplemented with 50 µg ml^−1^ DNase I (Merck/Sigma–Aldrich) and 1 mg ml^−1^ lysozyme (Merck/Sigma–Aldrich). After 1 h incubation while stirring, cell debris was spun down at 39 000*g* for 30 min. The supernatant was heated to 70°C while gently shaking, followed by another round of centrifugation at 4°C. Ammonium sulfate was added to the supernatant to a final concentration of 52.5%(*w*/*v*). This suspension was centrifuged at 39 000*g* for 1 h at 4°C and the pellet was resuspended in 2 ml purification buffer (20 m*M* HEPES pH 7.5, 300 m*M* NaCl). Overnight dialysis against the purification buffer was used to remove the remaining ammonium sulfate. The dialyzed sample was concentrated to approximately 0.9 ml, with a 50 kDa cut-off Vivaspin 2 Ultrafiltration concentrator (Sartorius) and ran on a size-exclusion Superose 6 10/300GL column (GE Healthcare Life Sciences) equilibrated in the purification buffer. The peak fractions containing pure apoferritin were concentrated and stored at −80°C after flash freezing in liquid nitro­gen. For cryo-EM grid preparation, purified apoferritin at a concentration of approximately 4 mg ml^−1^ was centrifuged at 50 000 rev min^−1^ for 10 min at 4°C to remove aggregates. A small amount of sample (3 µl) was applied to 300 mesh UltrAuFoil holey Quantifoil R 2/2 grids that were glow-discharged for 1 min in an ELMO glow discharge system (Cordouan). Grids were blotted for 4 to 6 s with the offset of −1 mm and humidity of 98% at room temperature, after which, they were immediately plunged into liquid ethane using Cryoplunge 3 (Gatan).

### 2.6. Single-particle data collection and image processing   

Images of apoferritin were collected on the CRYO ARM 300 electron microscope at a nominal magnification of 80 000, corresponding to a calibrated pixel size of 0.603 Å. The microscope illumination conditions were set to spot size 7, alpha 1, the aperture diameters for condenser and objective apertures were 100 and 150 µm, respectively. The energy filter slit was centred on the zero-loss peak with slit width set to 20 eV. Four images per hole were collected off-axis with a beam-image shift of 0.55 µm. Each micrograph was recorded as a movie of 80 frames with 0.1 s exposure time at a dose rate of ∼2 e^−^ pixel^−1^ s^−1^ (corresponding to a dose rate per frame of 0.6 e^−^ Å^−2^) resulting in a total dose of 48 e^−^ Å^−2^. Coma-free alignment by CTF was performed in *SerialEM* just before starting data collection. A total of 5377 micrographs were collected with a defocus range between −0.5 and −1.5 µm. After micrograph inspection 2794 micrographs were retained for further processing. The micrographs were selected based on the following criteria: total drift below 50 Å (calculated by integrating the trajectory of the frames) and early drift below 6 Å. Only micrographs with defocus between −0.2 and −1.5 µm were retained to ensure minimal contrast needed for particle alignment and to avoid CTF aliasing (Penczek *et al.*, 2014 ▸). A total of 100 805 particles were automatically picked with *crYOLO* (Wagner *et al.*, 2019 ▸). After 2D classification, 89 384 particles were selected for 3D reconstruction. 3D refinement was performed in *RELION* (v. 3.0; Zivanov *et al.*, 2018 ▸). The first run of the refinement resulted in a resolution of 2.5 Å, which increased to 2.4 Å after CTF and beam tilt refinement. The refined beam tilt was 0.29 mrad. To further correct for off-axis coma (Glaeser *et al.*, 2011 ▸), the dataset was split into subsets, each corresponding to one of the four directions of beam-image shift. The beam tilt was refined for each subset separately (Table S1 of the supporting information). Refinement for each subset independently resulted in a resolution between 2.2 and 2.3 Å. Following polishing and subset merging, the resolution further improved to 2.1 Å. Refinement of *C*_s_ from a nominal value of 2.7 to 2.55 mm resulted in further improvement in resolution to 1.9 Å (1.94 Å). To verify a possible influence of the cFEG flashing on the stability of beam tilt, the dataset was split into subsets acquired with the same beam shift and between two consecutive gun flashes. The beam tilt was refined separately for each subset and the deviation from the average tilt associated with the corresponding direction of beam-image shift was estimated by the refine CTF routine in *RELION* (v. 3.0). However, this did not increase the resolution. Further aberration correction in *RELION* (v. 3.1; Zivanov *et al.*, 2020 ▸) resulted in only minor resolution improvement to 1.86 Å. The final map, together with unmasked half-maps and the mask have been deposited in the EMDB under code EMD-10675 and the raw movies are deposited in EMPIAR with the accession code EMPIAR-10408. Calculations of FSC between the EM map and model were performed using the structure of mouse heavy-chain apoferritin (PDB entry 3wnw) refined in *PHENIX* using real space refinement (Afonine *et al.*, 2013 ▸).

## 3. Results and discussion   

### 3.1. Characterization of microscope performance   

**Figure 1:**
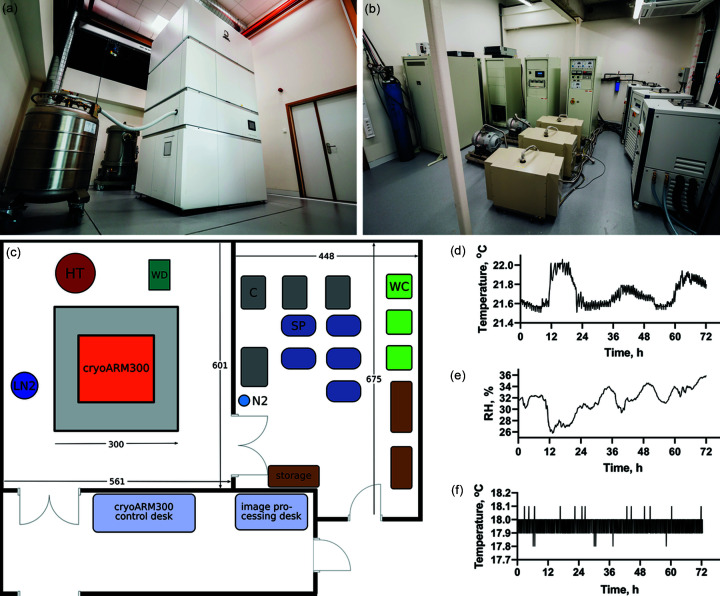
Microscope setup and environmental conditions for CRYO ARM 300 installed at BECM. Photographs of (*a*) the microscope and (*b*) technical rooms. (*c*) Schematic of the room layout and placement of the microscope with its supporting elements. Identical elements are indicated by identical colours. The grey square under the microscope indicates the isolated concrete slab. The following abbreviations are used: LN2, liquid nitro­gen tank; HT, high tension tank; WD, cooling water distribution unit; C, microscope electronics console; SP, scroll pump; WC, water chiller (two water chillers are used for JEOL JEM-1400 microscopes installed in the other rooms of the facility). Dimensions are indicated in centimetres. (*d*), (*e*) and (*f*) Air temperature and air relative humidity in the microscope room and temperature of cooling water plots during acquisition of the apoferritin dataset used for benchmarking. The 0.1°C wide band on the water temperature plot arises from regular water temperature fluctuations with a period of around 30 min. Data were recorded using Wi-Fi temperature and humidity loggers (FilesThruTheAir). The sensor for the microscope room data is located in the microscope room, outside of the microscope enclosure. Water temperatures were measured inside the secondary chiller tank.

The microscope room and stability of the environmental conditions are important determinants of the microscope performance; therefore, we provide a brief description of the setup of our microscope below. The CRYO ARM 300 is installed on an isolated concrete slab in a temperature- and humidity-controlled room [Figs. 1(*a*) and 1(*b*) ▸]. The layout of the room with dimensions and positions of the individual components is shown in Fig. 1 ▸(*c*) ▸. The temperature in the microscope room is maintained within a fork of 0.4°C [Fig. 1 ▸(*d*) ▸], whereas relative humidity is maintained below 40% [Fig. 1 ▸(*e*) ▸]. The technical room is temperature controlled only. Furthermore, the microscope room is equipped with an electromagnetic interference (EMI) cancellation system (JEOL). Cooling water is supplied to the microscope via a primary chiller, which creates a periodic temperature fluctuation with an amplitude of 3.5° and a period of around 30 min. This causes ±0.1°C fluctuation of the secondary cooling water temperature [Fig. 1 ▸(*f*)] which is used to thermostat the microscope lenses.

The temperature fluctuations influence the stability of the energy filter, which is otherwise very stable. The periodic fluctuations of the cooling water temperature result in periodic movements of the energy filter slit shadow with an estimated amplitude of between 2 and 3 eV (Fig. S1 of the supporting information). For routine data collection performed with a 20 eV wide well centred slit, the fluctuations of the slit position do not interfere with data collection.

The microscope was installed with a K2 detector, which was used for most of the experiments described in this article. It was replaced by K3 in late 2019 and images for some experiments were acquired on a K3 detector.

#### 3.1.1. Stability of electron emission by cFEG   

The CRYO ARM 300 is equipped with a cold field emission gun (cFEG). The lower energy spread of electrons of 0.3–0.4 eV emitted by cFEG versus Schottky FEG, 0.8 eV, results in a better temporal envelope function at spatial frequencies exceeding 0.5 Å^−1^, as follows from theoretical estimations (Wade & Frank, 1977 ▸). The beam intensity of cFEG decays over a period of a few hours due to contamination, and the intensity is recovered by a procedure of gun flashing that consists of turning off the extraction voltage, applying a few seconds long intense current through the filament to desorb contaminating molecules, followed by ramping up the extraction voltage to its nominal value. The gun flashing procedure is fully automated (it can be executed by software for automated data collection at the required moment) and it takes less than 2 min.

**Figure 2:**
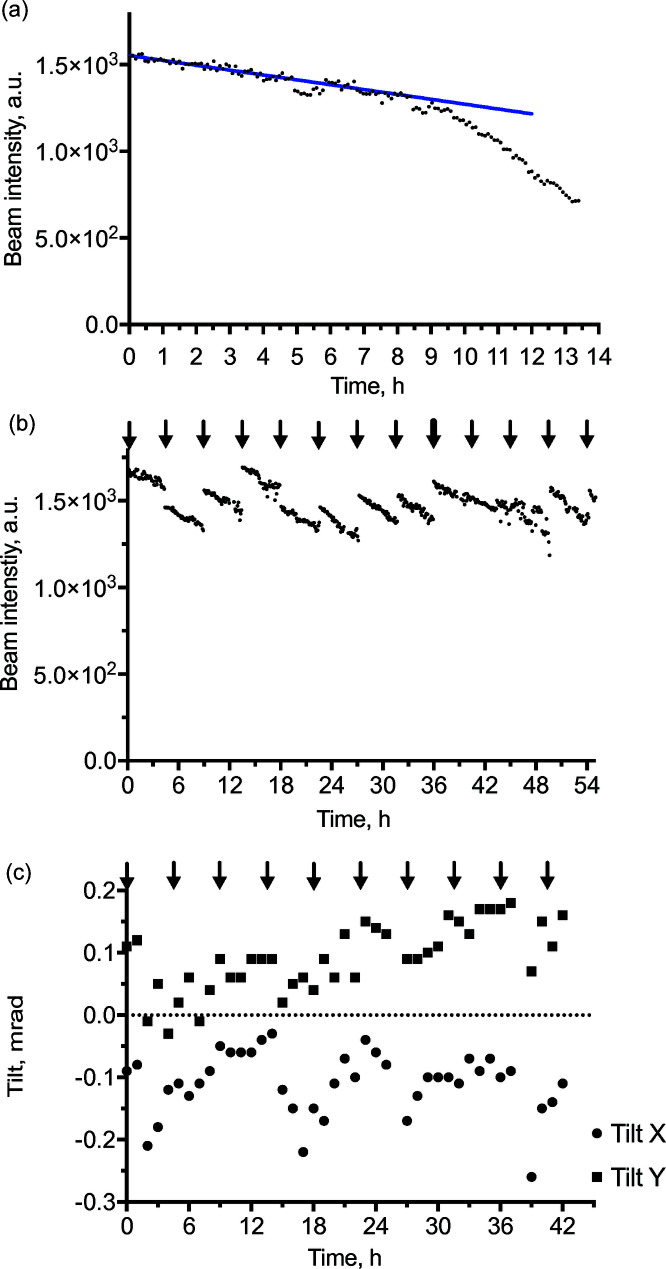
Characterization of cFEG stability. (*a*) Decay of electron beam intensity after HIGH gun flash. (*b*) Intensity changes of electron beam measured over a typical period of data collection with application of LOW gun flashes every 4.5 h as indicated by arrows and one HIGH flash, indicated by a thick arrow. (*c*) Electron beam tilt measured during an experiment shown in panel (*b*).

The beam intensity behaviour has already been characterized elsewhere (Hamaguchi *et al.*, 2019 ▸). In line with this study, we observed two-phased beam intensity decay after a HIGH gun flash: an initial linear phase with a decay rate of ∼2% per hour over the first *ca* 9 h, followed by a second phase with a faster decay rate [Fig. 2 ▸(*a*)]. Under the assumption that flashing recovers intensity, we applied LOW flashes (flash settings: current 1.4 Å over 0.7 s) every 4.5 h aiming to maintain electron flux variation within 10%. The resulting beam intensity recorded over a period of a 55 h corresponding to the duration of a typical dataset collection is shown in Fig. 2 ▸(*b*). We observe that the LOW flash produces somewhat unpredictable changes in the beam intensity resulting in intensity variation of up to 25% within the 55 h time period. Not even a HIGH flash (flash settings: current 2.8 Å over 1.4 s) recovered the beam intensity to the initial level. While this article was under revision, JEOL engineers changed the settings of the LOW flash to make it identical to the HIGH flash following the report by Hamaguchi *et al.* (2019 ▸). The corresponding behaviour of the beam intensity over a period of several flashes is shown in Fig. S2. The beam intensity did not recover to its original level after gun flashing. In this particular experiment, the amplitude of the intensity variation was around 17% which is a small improvement compared with LOW flash settings and might be a result of a shorter experiment. It can be clearly seen that whether HIGH or LOW flashes are used, the beam intensity not only fails to recover over several consecutive flashes, but occasionally drops upon flashing (Figs. 2 ▸ and S2).

Hamaguchi *et al.* (2019 ▸) observed large differences in the cFEG intensity fluctuations between regularly applied LOW and HIGH flashes. On our microscope, both flash settings result in approximately similar fluctuations of the beam intensity. The differences are probably caused by the difference in time-span between flashes. In our protocol, gun flashes are applied with a periodicity of 4.5 h, whereas Hamaguchi *et al.* applied flashes after a period of *ca* 8 h or longer. Therefore, it is possible that LOW flashes failed to desorb larger amounts of accumulated contaminants. However, other factors including specific differences between cFEG filaments and levels of vacuum in the cFEG chamber may affect the amplitude of the beam intensity fluctuations.

The beam intensity fluctuations lead to fluctuations in the electron dose between different cryo-EM images. The resulting fluctuations of signal-to-noise ratio (SNR) between the images are accounted for by maximum-likelihood refinement algorithms, like those implemented in *RELION* (Scheres, 2012 ▸), which estimate SNR for particle groups extracted from individual micrographs. However, the fluctuations of the electron dose are not accounted for by dose-weighted radiation-damage correction algorithms (Grant & Grigorieff, 2015 ▸; Scheres, 2014 ▸; Zheng *et al.*, 2017 ▸) resulting in sub-optimal correction of radiation damage and, as a consequence, sub-optimal 3D reconstruction.

The intensity fluctuations are a technical problem which can most likely be solved in the near future. Otherwise, special care should be taken at the image-processing step to account for the electron dose fluctuations. This in turn requires knowing the electron dose for each recorded image. Here we propose two approaches for doing this. First, to perform regular measurements of the blank beam intensity during data collection. The beam intensity is proportional to the electron dose and will allow estimation of relative electron dose at regular time intervals, between which the values can be interpolated. It might even be sufficient to measure the blank beam intensity only once between consecutive gun flashes since the intensity decay between the flashes appears to be reproducible (Figs. 2 ▸ and S3) and can be tabulated. The disadvantage of this approach is that it relies on the presence of an empty area on the grid, which is usually but not always the case. It may also require regular large stage movements that can have adverse effects on the data collection.

Alternatively, blank-beam intensity and consequently electron dose can be estimated during data collection if ice thickness measurements are performed regularly using the energy filter method (Rice *et al.*, 2018 ▸). Theoretically, the blank beam intensity, *I*_0_, can be determined from the following equation: 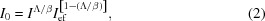
where *I* and *I*_ef_ are the beam intensities without and with the slit measured in the same position on ice, Λ and β are the apparent mean-free path of electrons in ice, and the aperture- and energy-filter limited scattering (Rice *et al.*, 2018 ▸), respectively, see Appendix *A* [App appa] for details. Such a method, if it works, would not be associated with additional time overheads. The anticipated difficulties here are the possible dependence of the coefficients Λ and β on sample composition and particle density, as well as on the presence of additional support films.

The stability of the beam tilt is critical for high-resolution 3D reconstruction (Glaeser *et al.*, 2011 ▸; Zivanov *et al.*, 2018 ▸); therefore, we assessed the beam alignment stability upon cFEG flashing. The beam position remained stable within a few nanometres upon flashing while the amplitudes of the beam tilt in the *X* and *Y* directions were 0.23 and 0.21 mrad, respectively, with a standard deviation for each direction of 0.05 mrad [Fig. 2 ▸(*c*)]. Interestingly, the gun flashing does not seem to affect the beam tilt *per se*, since there are no jumps in the beam tilt upon gun flashing. The beam tilt does however change gradually over the time period of 42 h, which may indicate instabilities caused by environmental factors. The observed deviations of ±0.1 mrad are not significant enough to affect 3D reconstruction to a resolution up to around 2 Å.

One of the consequences of the improved cFEG beam temporal coherence is enhancement of diffraction fringes produced by the edges of the defocused condenser aperture (Fig. S3). The fringes spread over a distance of at least 150 to 200 nm from the edge of the aperture, rendering this area unusable for imaging. Consequently, for common imaging conditions with a magnified pixel size of ∼1 Å and a corresponding diagonal of the K2 detector of 530 nm, the required minimal beam diameter is 900 nm, making only 22% of the total beam area suitable for imaging. Thus, the strong fringes reduce the area suitable for single-particle data acquisition within individual holes and consequently reduce the rate of data collection. The extension of fringes can be reduced or fringes can be almost completely eliminated using Kohler illumination (Benner & Probst, 1994 ▸). It was possible to eliminate the fringes by either adjusting condenser illumination settings, or shifting the *Z* height of the stage. Under these conditions, however, the largest illumination area attainable in Search mode of the minimal dose settings was limited by the condenser aperture to an area too small to enable reliable stage centring on the selected holes. Although it was possible to retrieve the condenser aperture during the search procedure, we decided not to use this option as a part of the standard data acquisition protocol due to a limited reproducibility of the aperture positioning after hundreds of cycles of aperture insertion.

#### 3.1.2. Stage properties   

Sample stage stability is one of the key factors that define the suitability of a microscope for high-throughput automated data collection; therefore, knowledge of stage drift induced by regularly performed procedures and during automated data collection is important for evaluating the performance of the instrument.

The drift rates measured in this work were derived by analysing the trajectory of the movie frames aligned by *MotionCor2*. Initially we estimated drift by summing the distances of shifts between individual frames and dividing by the total exposure time. However, we realized that, for conditions with short exposure times per frame, this approach results in overestimated drifts, which are not consistent with our knowledge about the stage properties. We think this overestimation is a result of the accumulation of frame alignment errors that depend on many factors including image contrast, program parameters and probably some others. On the other hand, after a few seconds of stabilization delay, the residual stage drift is expected to be linear. In this case, measuring the distance between the first late exposure frame and the last frame of the movie produces a more accurate measurement of the stage drift. When measured with this method, stage drifts are consistent between data acquired with different frame rates (Fig. S4), while integration of the trajectory results in drift rates that grow with increasing frame rate. Therefore, here we used the method based on measuring the distance between the first late and the last frames to estimate the stage drift excluding drifts measured after liquid nitro­gen refilling, where the drift pattern appeared more complex than a continuous linear drift.

**Figure 3:**
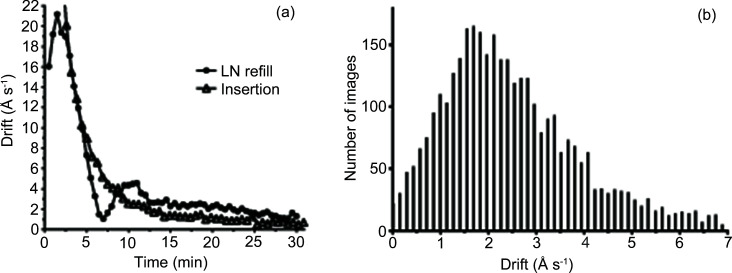
Stage stability of the CRYO ARM 300. (*a*) Sample drift after completion of nitro­gen refill in both stage and autoloader dewars (filled circles) calculated by the integral path method and after cartridge insertion from the autoloader into the microscope column (open triangles) calculated by the end-to-end method. (*b*) Histogram of stage drift for images recorded during data collection of apoferritin. Drift is measured by the end-to-end method.

The procedures of sample insertion in the microscope column and refilling of the liquid nitro­gen (LN) dewars induce strong stage drift that decreases over time. The time needed to stabilize the drift is subtracted from the total time available for data collection and therefore should be as short as possible. We measured the drift of the stage after grid insertion in the column from the autoloader and after refilling of the LN dewars [Fig. 3 ▸(*a*)]. In both cases, the drift reduces to *ca* 2 Å s^−1^ within around 15 min.

This results in a loss of around 30 min per 24 h of data collection, since dewar refilling occurs every 12 to 14 h. The grid insertion is usually followed by acquisition of a grid atlas recorded at low magnification which is much less sensitive to stage drift. In this case, 3 min equilibration time is sufficient.

We also assessed the reproducibility of the grid position after grid retraction into sample storage and re-insertion back onto the microscope stage. The average position offset after re-insertion was found to be 2 µm (*n* = 4) with a maximum observed shift of 4.5 µm. These shifts are easily corrected by adjusting registration which enables reliable automated data collection from the preselected positions on multiple EM grids. It should be noted that, due to the grid cartridge design in the shape of an elongated metal bar (5.00 × 33.00 × 1.30 mm, *W* × *L* × *H*), the differences in grid rotation upon re-insertion are negligible and did not cause noticeable atlas misalignment after minor adjustment of the registration by shift.

Furthermore, we assessed the stage drift during single-particle data collection of apoferritin. In the procedures used at our microscope, mechanical positioning of the stage takes on average 48 s (*AlignToItem* procedure of *SerialEM*) (Table 1 ▸) followed by an additional stabilization time of 5 s and recording of four micrographs per stage position. The resulting distribution of drifts for the complete dataset with obvious outliers (which represent images without ice, a few images recorded on gold and images with too-low defocus which failed to align) removed is shown in Fig. 3 ▸(*b*). The distribution of drift rates has a peak around 2 Å s^−1^ with 90% of images having drift rates below 4.4 Å s^−1^. For the apo­ferritin dataset it translates into an average drift per frame of below 0.44 Å. For most of other datasets collected, the distribution of drift peaks around 2 Å s^−1^, with 90% of drift below 4 Å s^−1^ (Fig. S4). The stage behaves in a similar manner during collection of single-particle data with the stage tilted to 40° [Fig. S4(*d*)]. For data recorded on the K2 detector the typical exposure time per frame was 0.2 s, which translated into an average drift per frame of ∼0.4 Å and maximum of 0.8 Å. Such a stage drift does not have significant influence on the quality of the micrographs to a resolution of at least 2 Å (see Appendix *B* [App appb] and Fig. S6). With faster cameras exposure time per frame is further reduced, thus with a K3 camera, characteristic exposures per frame are on the order of 0.05 s. Therefore, drifts up to 10 Å s^−1^ or even higher can be tolerated without significant deterioration of data quality. We can conclude that the CRYO ARM 300 cryo-stage is stable enough for routine high-resolution single-particle cryo-EM data collection.

#### 3.1.3. Ice contamination   

**Figure 4:**
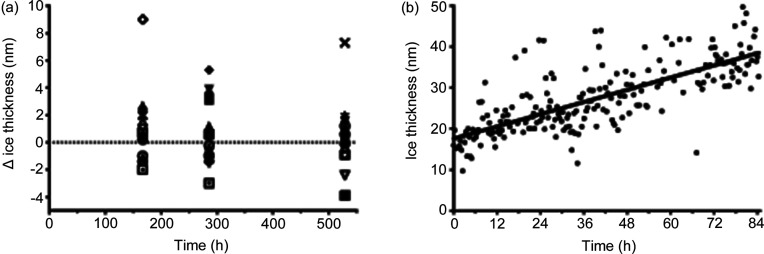
Ice contamination rates. (*a*) Ice thickness changes for a grid stored in the autoloader. (*b*) Ice thickness measured during data collection of the apoferritin dataset. Obvious outliers were removed. The linear fit has a slope of 0.25 nm h^−1^ and starting value of 17.6 nm.

Long-term preservation of cryogenic samples during sample screening, storage and data collection is an indispensable requirement for a modern automated electron microscope. Ice contamination is the major reason for deterioration of cryo-EM samples. We estimated the ice contamination rate for the samples kept in the autoloader by measuring the ice thickness in selected holes of a cryo-EM grid immediately after insertion into the microscope and on days 7, 12 and 22 [Fig. 4 ▸(*a*)]. The corresponding average increases in the ice thickness were 1.1 ± 0.6, 0.4 ± 0.5 and 0.2 ± 0.6 nm (*n* = 15). The total electron dose applied per measurement, including grid positioning, was below 0.5 e^−^ Å^−2^. Hence, the electron-induced ice thinning was below 1 Å (Wright *et al.*, 2006 ▸) and cannot account for low ice contamination. The absence of measurable contamination is surprising; however, it is consistent with cryo-EM images of the grids taken 22 days apart (Fig. S5), which display no significant differences. We conclude that cryo-EM grids can be kept in the autoloader for a period of at least two weeks without noticeable sample deterioration. This property is valuable for collecting data from multiple grids and management of multiple cryo-EM projects.

To estimate the ice contamination rate in the microscope column, the automated ice thickness measurement procedure using the energy filter method (Rice *et al.*, 2018 ▸) was introduced in the *SerialEM* script. It is noteworthy that this method relies on two measurements of image intensity taken with inserted and removed energy slits which are done immediately after each other; therefore, the resulting ice thickness measurements are not affected by slow decay of cFEG intensity. The ice-thickness values measured for a subset of images recorded during collection of the apoferritin dataset (see below) are shown in Fig. 4 ▸(*b*). The gradual increase in the ice thickness can be clearly seen despite the areal spread of the data points. The linear fit of the ice thickness against time indicates an ice growth rate of *ca* 2.5 Å h^−1^, or 6 nm per 24 h. This value is within the JEOL specifications for an in-column ice contamination rate of 5 Å h^−1^. Such an ice contamination rate is not negligible and should be taken into account when data are collected over periods of time exceeding two days from samples with very thin ice and particularly for small particles. However, for the more commonly encountered conditions for which the ice thickness exceeds 50 nm the effect of the ice contamination is not significant. Moreover, with the increasing rate of data collection due to faster detectors and the use of coma-corrected imaging (Wu *et al.*, 2019 ▸), the duration of the data collection rarely needs to be longer than 48 h, making the negative impact of ice contamination less important.

### 3.2. Automated data collection   

Automated data collection on our microscope is performed using *SerialEM* (Mastronarde, 2005 ▸). The initial scripts provided by JEOL application scientists were significantly modified and are provided in the supporting information. Among the main modifications are the introduction of sparse focusing, regular ice thickness measurements using an energy filter and automated grid atlas acquisition.

The timing for individual steps of the data-collection procedure is summarized in Table 1 ▸. Our data-collection procedure includes regular beam centring. The beam position during data collection deviates from the average position with an amplitude up to 100 nm. One constituent of this deviation correlates with temperature changes in the enclosure induced by liquid nitro­gen tank refilling that has an amplitude of up to 25 nm and lasts several hours, while the nature of the other contributing components has not yet been identified and might be caused by environmental factors.

The throughput of data acquisition using a K2 direct electron detector is between 40 and 100 images per hour for one to four images recorded per stage position. This corresponds to a daily throughput of between 1000 and 2400 images. As a standard setting, the images are recorded with the energy filter slit set to 20 eV. The microscope is stable enough to reliably collect data over a period of several days with minimal supervision and no need for microscope re-alignment.

In a routine data-collection cycle four grids are loaded into the microscope and the grid atlases are collected. This procedure takes about 1 h. After imaging of a few selected holes to verify ice thickness and particle distribution, square atlases are acquired and positions for data collection are selected. Coma-free alignment is performed just before the launch of the data collection. The preparatory steps before the launch of data collection, including grid clipping and transfer to the microscope, screening and hole selection, take about 4–5 h. If necessary, more grids are loaded and screened on the same day or a more thorough search for the optimal imaging locations on the grid is performed on the same day.

Due to the lack of data collection time on high-resolution microscopes, pressure for increasing the data collection throughput is ever higher. A number of further optimizations of the data collection protocol are possible to increase the throughput. Currently the stage positioning time is relatively long because up to three alignment cycles are being used in *SerialEM* for stage positioning with an accuracy of 0.3 µm. These settings can be optimized to reduce the stage alignment time. Faster detectors will reduce image acquisition time. Our preliminary estimates show that on a K3 detector similar collection settings can reduce the image recording time from 20 to around 6 s. Data collection throughput can be further significantly increased by introducing aberration-corrected beam-image shift conditions to record images from multiple holes without moving the stage (Wu *et al.*, 2019 ▸).

### 3.3. Benchmark with apoferritin   

**Figure 5:**
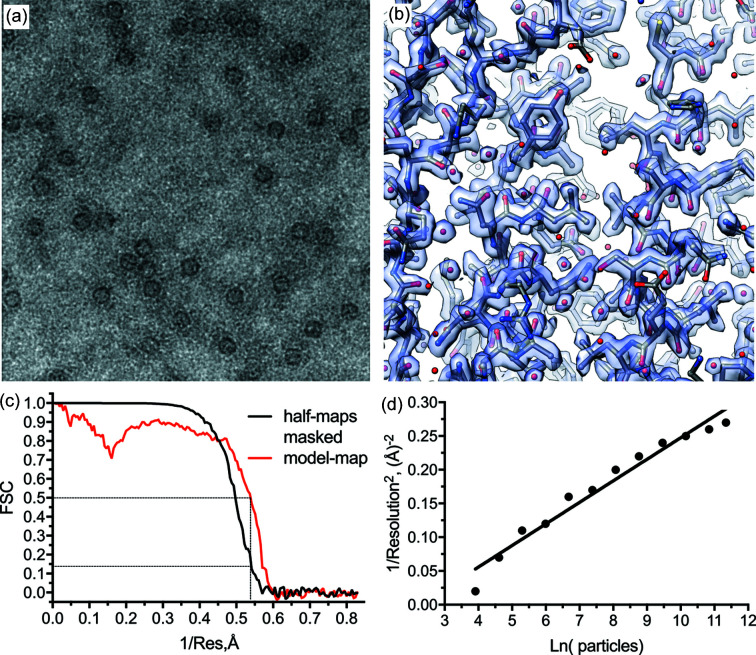
Benchmark with mouse heavy-chain apoferritin. (*a*) Typical EM image of the collected apoferritin sample. (*b*) Density map of the reconstruction at 1.9 Å resolution displaying characteristic features of the map. (*c*) FSC curves: gold standard masked half-maps (black) and map versus model (red). (*d*) *B*-factor plot (Rosenthal & Henderson, 2003 ▸) indicating a *B* factor of 62 Å^2^.

As a benchmark, we collected a dataset of mouse heavy-chain apoferritin. Our protein preparation produced cryo-EM grids with low particle density in ice [Fig. 5 ▸(*a*)]. Therefore, a dataset exceeding 5000 images was collected, resulting in a reconstruction to a resolution of 1.9 Å from around 90 000 particles [Table 2 ▸, Figs. 5 ▸(*b*) and 5(*c*)]. Apart from the higher magnification (80 000), this dataset was collected under settings used for regular data collection including a beam-image shift of 0.55 µm from the hole centre to record four images within 2 µm holes. To reach sub-2 Å resolution reconstruction the *C*_s_ value was refined from the nominal value of 2.7 to 2.55 mm. The deviation of the refined values of *C*_s_ from the nominal values specified by the manufacturer have also been observed for other microscopes and can indicate specific properties of the particular objective lens as well as incorporate other systematic changes of experimental conditions including altered accelerating voltage (Wu *et al.*, 2020 ▸).

The average residual beam tilt refined in *RELION* for the single-particle data was 0.3 mrad (Table S1), which is a significant deviation from coma-free conditions and occurred despite the coma-free alignment performed before launching the data collection. This appears to be a systematic issue and needs further investigation. Furthermore, we found that the beam-image shift of 0.55 µm induced 0.2 mrad beam tilt (Table S1) which had to be corrected for to reach sub-2 Å resolution. This is significant enough to affect the phases of 3D reconstruction below 2.8 Å resolution (Glaeser *et al.*, 2011 ▸). Estimated in such a way the beam-shift induced coma on the CRYO ARM 300 is about twice as high as the values reported for the Titan Krios microscope, *i.e.* 0.36 versus 0.19 mrad µm^−1^ for the beam-image shift, respectively (Cheng *et al.*, 2018 ▸; Zivanov *et al.*, 2018 ▸). This can be due to differences in optics between two microscopes, or not accurate enough alignment of pivot points in spite of the fact that they were carefully aligned following a procedure recommended by JEOL.

The *B* factor of 62 Å^2^ calculated for the dataset [Fig. 5 ▸(*d*)] (Rosenthal & Henderson, 2003 ▸; Zivanov *et al.*, 2018 ▸) is comparable to data collected on the Titan Krios microscope (Zivanov *et al.*, 2018 ▸). It is worth noting that a smaller pixel size was used for the CRYO ARM 300 dataset compared with the dataset acquired on Titan Krios, 0.603 versus 0.814 Å, respectively. Hence, due to higher oversampling in the former case, the effective DQE of the K2 detector (Ruskin *et al.*, 2013 ▸) is more favourable for the CRYO ARM 300 dataset than for the Titan Krios dataset (Stagg *et al.*, 2008 ▸). The somewhat curved distribution of points in the *B*-factor plot [Fig. 5 ▸(*d*)] suggests that higher order aberrations have not been adequately corrected. This might be caused by coma induced by beam-image shift applied to align the beam to the hole centres, inducing beam tilts of up to 0.1 mrad which affects resolution starting from 2.2 Å (Glaeser *et al.*, 2011 ▸). Due to the low number of particles per micrograph, the efficiency of refinements including per particle CTF refinement, Bayesian particle polishing and per micrograph beam-tilt refinement – which all rely on high particle density – were probably not very efficient, limiting the final resolution of the refinement compared with other very high-resolution reconstructions of mouse heavy-chain apoferritin obtained with CRYO ARM 300 (Kato *et al.*, 2019 ▸). Nevertheless, despite the low particle density, sub-2 Å resolution reconstruction was still possible. Further improvement in resolution can likely be achieved by collecting data without applying beam-image shifts or using image-shift induced coma correction.

**Figure 6:**
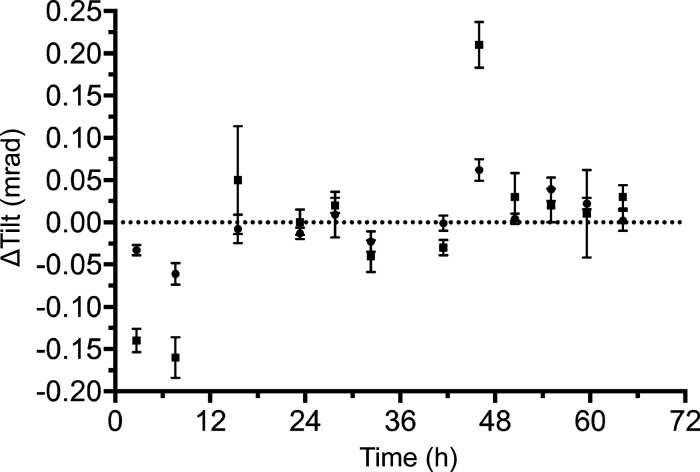
Beam tilt stability during data collection. Beam tilt relative to the average beam tilt of the dataset is shown for subsets of data collected between gun flashes. Circles, tilt along the *x* axis; squares, tilt along the *y* axis.

Furthermore, refinement of the beam tilt (Zivanov *et al.*, 2018 ▸) allowed us to compare the beam tilt between gun flashes during the actual data-collection process (Fig. 6 ▸). The plot indicates that the average beam tilt between gun flashes is stable within 0.1 mrad resolution and, hence, preserves the resolution to better than 2.2 Å (Cheng *et al.*, 2018 ▸; Glaeser *et al.*, 2011 ▸). The few outliers in Fig. 6 ▸ are likely to be the result of noisier reconstructions caused by the smaller number of particles in particular subsets.

### 3.4. Microscope usage statistics   

**Figure 7:**
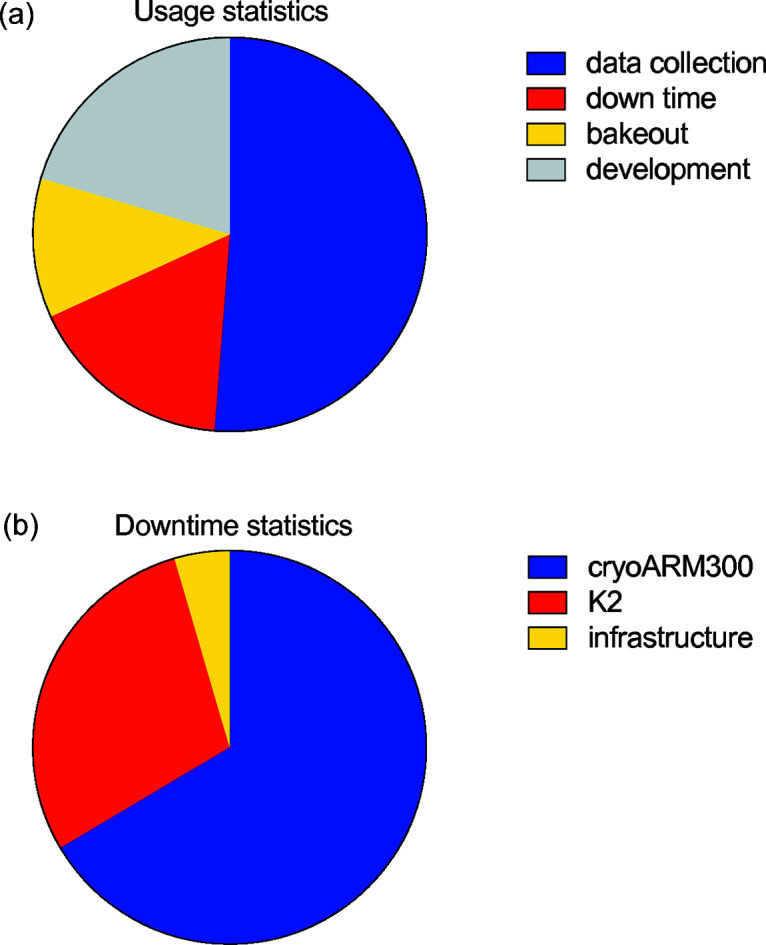
Microscope usage statistics during a period of six months of the facility operation. (*a*) Time split of microscope usage. (*b*) Statistics of downtime causes.

The long-term reliability of the microscope during continuous operation in multiuser settings can be assessed by analysis of the fraction of time used for data collection versus time used for regular maintenance procedures and technical downtime. Usage statistics of our CRYO ARM 300 over a period of six months is summarized in Fig. 7 ▸. A total of 72% of the time was used for data collection and development of the data-collection procedures. Regular two-day bakeout cycles performed every three weeks account for 11% of the total time. The remaining 17% of downtime was due to breakage of the microscope caused by minor technical problems with the CRYO ARM 300, the K2 detector and infrastructure (cooling water, air conditioning, air and nitro­gen supply) [Fig. 7 ▸(*b*)]. We could see a trend in decreased downtime over time, and during longer periods of microscope use we expect the time useful for data collection to surpass 80%. In a period of six months of constant operation 33 successful datasets were collected, accounting for around 96 000 images and 85 tomograms on 15 different projects.

## 4. Conclusions   

Here we characterized the suitability and performance of the CRYO ARM 300 with a K2 detector for automated single-particle cryo-EM data collection. We found that the beam intensity fluctuations of the cFEG due to gun flashing are significant but not detrimental for the quality of single-particle 3D reconstructions. We suggested possible approaches on how to monitor the beam intensity during the data collection which can be integrated into data collection procedure and later allow for more accurate correction of radiation-induced sample damage. We found that the microscope stage is stable enough to permit routine high-resolution automated data collection without the need for significant delays after stage positioning. The ice contamination rate is low enough to enable data collection over a period of several days without significant contrast losses. The contamination rate is particularly low in the autoloader, enabling sample storage for weeks without deteriorating the ice quality. Finally, *SerialEM* permits efficient and reliable data collection over long periods of time without the need for supervision. The routine data collection protocols deliver datasets that can be reconstructed to resolutions exceeding 2 Å. We conclude that the CRYO ARM 300 is suitable for repetitive reliable multiday data collection in the environment of a multiuser facility. It should also be noted that while cFEG does provide advantages over Schottky FEG for very high-resolution 3D reconstruction, as has been shown by the 1.54 Å reconstruction of apoferritin (Kato *et al.*, 2019 ▸), today such high resolution can be achieved only for a limited number of samples, but this may change in the future. Ultra-high-resolution reconstructions critically depend on very good microscope stability, in particular stability of the beam tilt, which still need to be improved on the CRYO ARM 300 before it can become an instrument for routine collection of ultra-high resolution single-particle cryo-EM data.

## Supplementary Material

EMDB reference: apoferritin, EMD-10675

SerialEM scripts for automated data collection on CryoARM300. DOI: 10.1107/S2052252520006065/pw5013sup1.zip

Supplementary figures. DOI: 10.1107/S2052252520006065/pw5013sup2.pdf

## Figures and Tables

**Table 1:** Timing of the steps of automated data collection in *SerialEM*

Data collection parameter	Time
Images per hole	1–5
cFEG flashing frequency	4.5 h
LN tank refilling frequency	12–14 h
Alignment of the stage to a hole position with 0.3 µm accuracy and up to 3 cycles	48 ± 8 s
Focusing (every 5–10 µm)	33 ± 5 s
Beam centering (every 3 min)	8 ± 5 s
Stage stabilization delay	0–15 s
Image recording on K2 (10 s exposure, 60 frames)	22 ± 1 s

**Table 2:** Data collection parameters and statistics for the apoferritin dataset

Data collection	
Electron microscope	CRYO ARM 300
Electron detector	K2
Voltage (kV)	300
Defocus range (µm)	0.5–1.5
Pixel size (Å)	0.603
Electron dose (e^−^ Å^−2^)	48
Images	2348
	
3D reconstruction	
Final particles	89384
Applied symmetry	O
Resolution (Å)	1.9
*B* factor (Å^2^)	43

## Appendix A Recovering blank beam intensity from ice thickness measurements

The ice thickness, *d*, can be determined experimentally by taking images with and without an energy filter slit at the same position using equation (1), or from the intensity of blank beam *I*_0_ and the intensity in the presence of sample without an energy slit using the aperture-limited scattering coefficient (Rice *et al.*, 2018 ▸),

From expressions (1) and (3), it is evident that the ice thickness can also be determined from values of *I*_0_ and *I*_ef_ as

where β is the aperture- and energy-filter limited scattering coefficient, defined as

Since the ice thicknesses determined using equations (1) and (4) are identical, then

Rearranging equation (6) we obtain an expression for *I*_0_:

The values of *I* and *I*_ef_ are measured experimentally during routine data collection and sparse ice thickness determination using the energy filter method. Using equation (7), the blank beam intensity can be determined and associated with the images recorded within a certain time frame after the ice thickness measurement, allowing for an accurate dose weighting during image processing.

## Appendix B Effect of drift on the resolution

The effect of drift on the envelope of EM images is easy to calculate if we assume that between individual frames the drift can be approximated by unidirectional movement with constant velocity. The mathematical expression for the resulting dumping function is well known, see for example the work by Buseck *et al.* (1989 ▸). For clarity, we provide the derivation of this expression below.

The drifted image, *I*_dr_, can be described by convolution of the image without drift, *I*, with top-hat function *T* of width *a*, where *a* is the drift between two consecutive frames:

For simplicity, we consider a 1D case. Following convolution theorem, the Fourier transform (FT) of such an image is a product of the FT of the image with the FT of the top-hat function, with *k* being the spatial frequency:

or in normalized form, such that *F*{*T*(0)} = 1:

The plot of *F*{*T*(*x*)} is shown in Fig. S6 for several representative values of drift per frame. The result of the drift is the attenuation of Fourier amplitudes of the image in the direction of the drift. The Fourier signal completely disappears at a resolution of *a*; at resolutions of 2*a* and 3*a* the amplitudes are attenuated to 63 and 82%, respectively.
