# Supplementary material for: Assessing the JEOL CRYO ARM 300 for high-throughput automated single-particle cryo-EM in a multiuser environment
Source: IUCrJ. 2020 Jun 11;7(Pt 4):707–18. doi: 10.1107/S2052252520006065 (PMC7340256; doi:10.1107/S2052252520006065)
Supplement: Supplementary file 2 [file m-07-00707-sup2.pdf]

IUCrJ

Volume 7 (2020)

Supporting information for article:

Assessing JEOL cryoARM300 for high-throughput automated single particle cryo-EM in multiuser environment

Marcus Fislage, Alexandr Shkumatov, Annelore Stroobants and Rouslan G. Efremov

Table S1 Refined beam tilt for off-axis images taken with beam-image shift of 0.55 µm in four directions during data collection of apoferritin.

Direction	Tilt X, mrad	Tilt Y, mrad	Total tilt, mrad	Deviation from average X, mrad	Deviation from average Y, mrad	Deviation from average total, mrad
90°	-0.34	0	0.34	-0.05	-0.11	0.12
180°	-0.23	0.11	0.25	-0.06	0.22	0.22
270°	-0.17	-0.26	0.31	0.12	-0.15	0.19
360°	-0.42	-0.28	0.50	-0.13	-0.17	0.21
Average	-0.29	-0.11	0.30			0.2

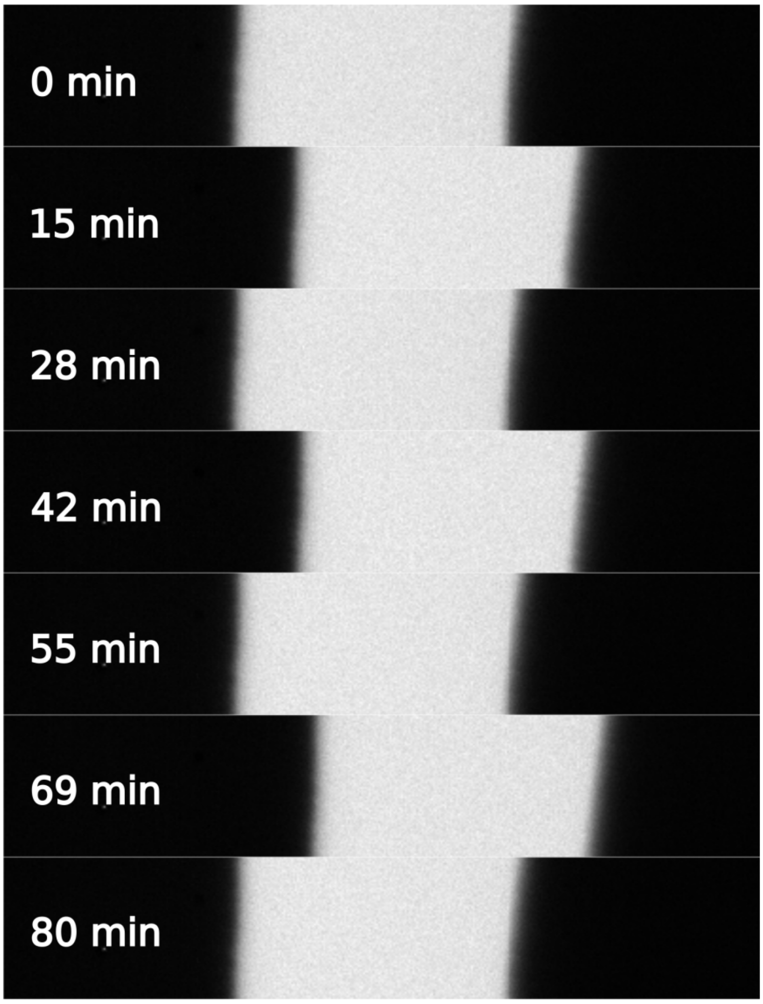

Figure S1 Fluctuations of the position of Ω-energy filter slit shadow induced by fluctuations of the cooling water temperature. The images of slit set to width of 10 eV were recorded at nominal magnification of 10,000 showing slit as a vertical bright line on the image were recorded every 10 sec. A total of 390 images were recorded on K3 detector with exposure of 1 s. Horizontal slabs from the same position of the image are shown for the time points displaying extreme positions of the filter slit. The shift was calibrated relative to the slit width. The average amplitude of movements is 2.7 eV (n = 4) and period of fluctuations is around 26 min.

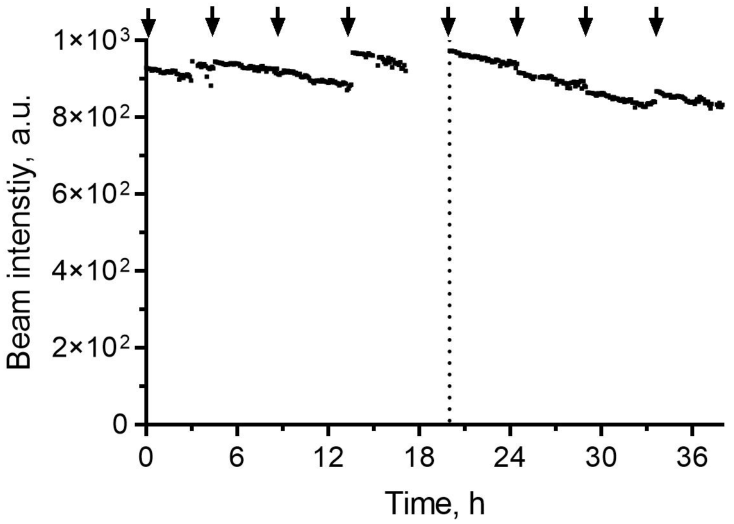

Figure S2 Intensity changes of electron beam measured over a period of several HIGH gun flashes applied with an interval of 4.5 hours are shown. The flashes are indicated by arrows. The data were recorded in two separate experiments conducted under identical conditions. The vertical dotted line separates the first from the second experiment.

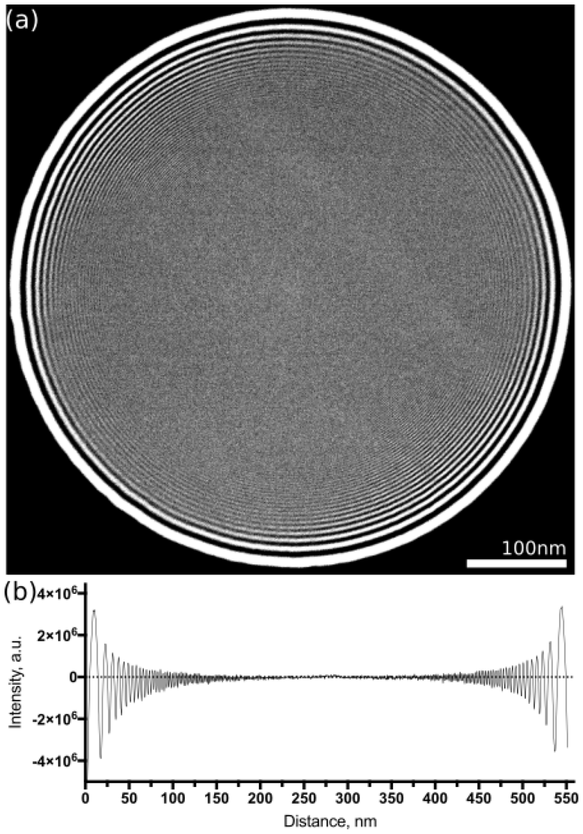

Figure S3 Diffraction fringes originating from condenser aperture. (a). Image of the beam recorded at magnification of 25,000 and condenser aperture of 70 um, alpha angle 1. (b) Radial profile of the beam intensity averaged along 100 parallel rows of pixels.

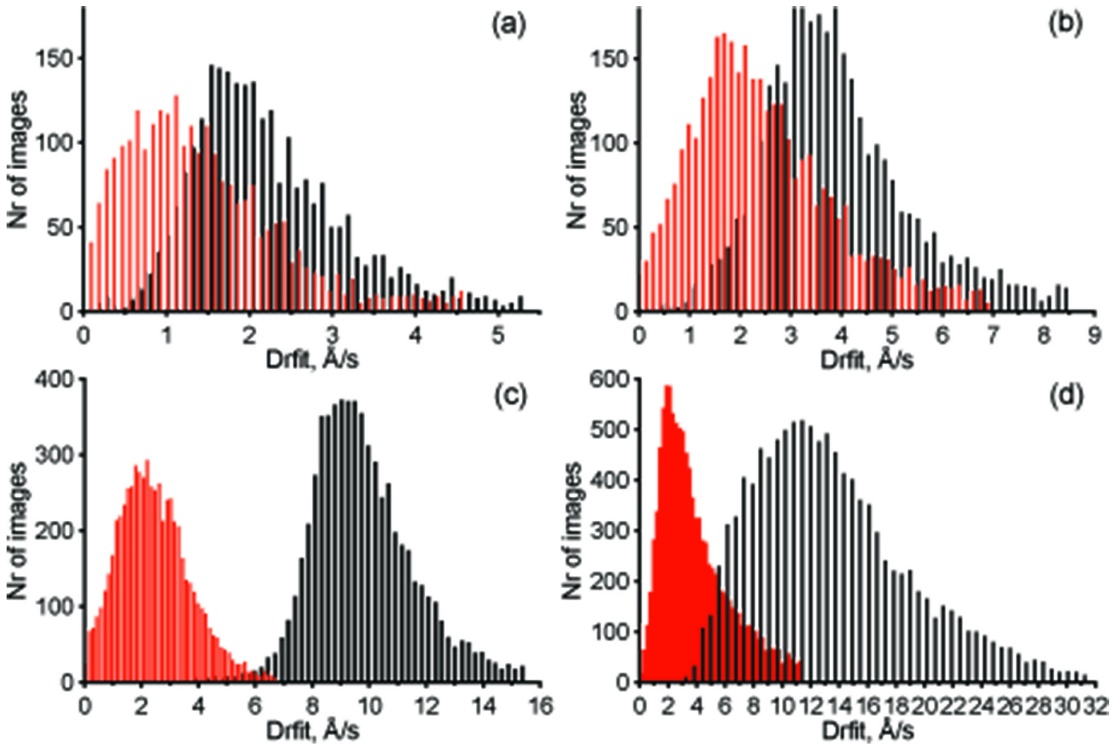

Figure S4 Comparison of two methods for calculating stage drift based on the trajectory of frame movement determined by MotionCor2. Histograms of image drifts are shown for single particle datasets collected at the following conditions: (a) K2 detector, 0.2 s/frame, 50 frames; (b) K2 detector, 0.1 s/frame, 80 frames; (c) K3 detector, 0.038 s/frame, 59 frames; (d) data collected with stage tilted by 40°, K3 detector, 0.038 s/frame and 59 frames. Black histograms show drift as calculated by trajectory integrating method, red – calculated by end-to-end method.

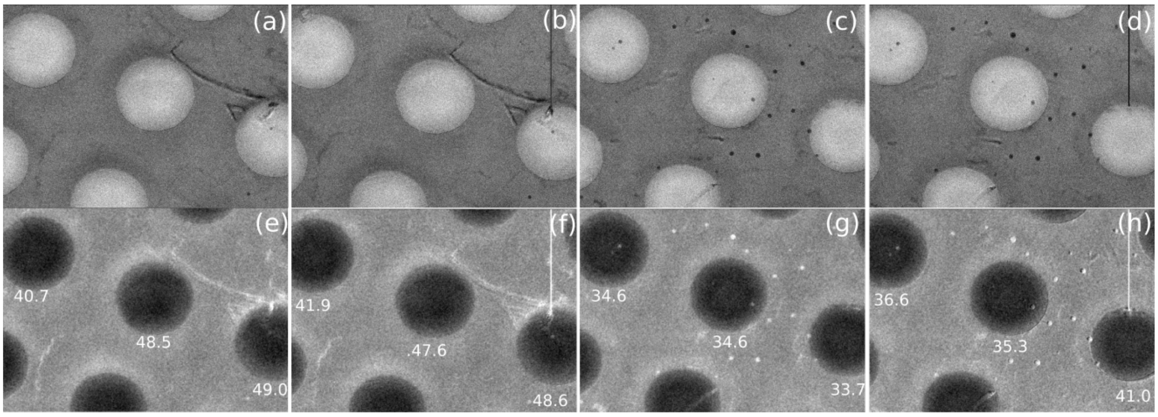

Figure S5 Ice contamination in the autoloader. Images of the same grid at two positions taken after grid insertion into the microscope (a), (c) and after 22 days of storage in the autoloader (b), (d). Panels (e-f) show corresponding images of ice thickness calculated using equation 1. The average ice thickness measured for the holes is indicated in nm.

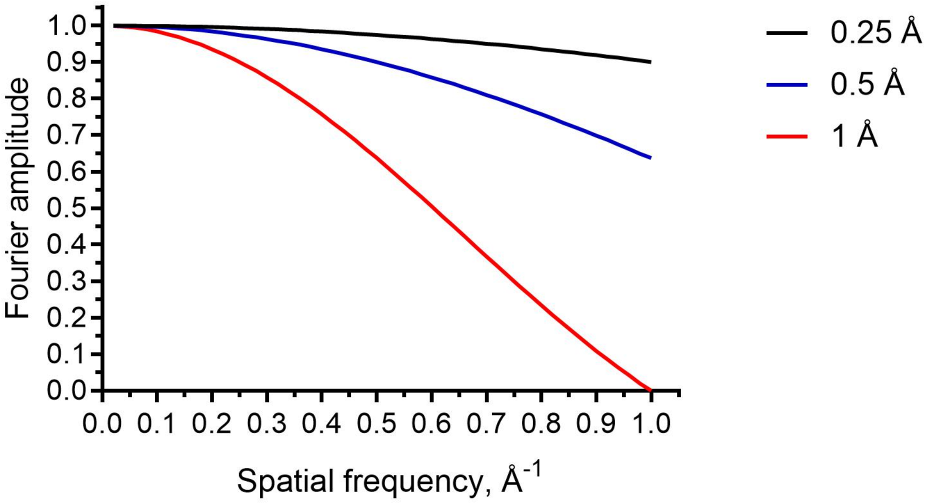

Figure S6 Drift-induced decay of Fourier signal shown for selected values of drift per frame as function of spatial frequency.
